# DeWitt County Medical Society

**Published:** 1869-05-15

**Authors:** John A. Edmiston

**Affiliations:** Sec’y.


### *De Witt County Medical Society.*

The society met in annual session at the office of Drs. Goodbrake & Edmiston, in the City of Clinton.

The President, Dr. J. A. Edmiston, in the Chair.

Minutes of last meeting read and approved.

Dr. W. G. Cochran, of Farmer City, was proposed for membership, and elected.

Officers for the ensuing year: President, Dr. John Wright; Vice President, Dr. J. W. Edmiston; Treasurer, Dr. C. Goobrake; Secretary, Dr. Edmiston; Censors, Drs. W. G. Cochran, C. Goodbrake, J. H. Tyler.

Dr. Wright, in a neat speech, returned thanks for the honor conferred upon him.

The retiring President, Dr. A. Edmiston, asked until next regular meeting to complete his annual address, which was, on motion, granted.

Drs. C. Goodbrake and John Wright were elected delegates to the American Medical Association, for the next year; and Drs. C. Goodbrake, J. Wright, and B. S. Lewis, were elected delegates to the Illinois Medical Society, with power to designate alternates in case of inability to attend.

Various subjects were brought before the meeting and discussed at length.

Drs. W. G. Cochran and J. H. Tyler were appointed essayists.

On motion, the Secretary was instructed to furnish copies of proceedings to the Clinton papers and Chicago Medical Journals, for publication.

On motion, adjourned until next regular meeting.

John A. Edmiston, *Sec'y.*

### ORIGINAL COMMUNICATIONS.

Leavenworth, April 10, 1869.

Dr. J. Adams Allen:

Dear Sir:—A person, calling himself Dr. M. F. Turner, is traveling through Kansas with a letter, purporting to have been written by yourself, recommending him to the good offices of medical men.

The letter looks like a forgery to me, and I am certain that the person, though a cripple, is unworthy of the aid he is asking. If you will inform me, if the letter is not genuine, I will put the medical men of the State upon their guard against the man. Very truly, yours,

C. A. Logan.

---

Ann Arbor, Mich., March 28, 1869.

Dr. J. A. Allen:

Dear Sir:—J. W. Shipton, M.D., class of 1867-8, of Rush Medical College, died in Detroit, March 19, 1869.

He was on his way from Florida to his home in Elizabeth, Ill., when he was taken suddenly worse, and died in about four hours. He had been South for the benefit of his lungs.

You may receive more particulars from other sources, but thought I would communicate the fact, as no one else might do so.

Yours, etc.,

J. T. Scovell, Class 1867-8, R. M. C.

